# High-Reliability Health Care: Getting There from Here

**DOI:** 10.1111/1468-0009.12023

**Published:** 2013-09-13

**Authors:** Mark R Chassin, Jerod M Loeb

**Affiliations:** The Joint Commission

**Keywords:** quality improvement, patient safety, safety culture, high reliability

## Abstract

**Context:**

Despite serious and widespread efforts to improve the quality of health care, many patients still suffer preventable harm every day. Hospitals find improvement difficult to sustain, and they suffer “project fatigue” because so many problems need attention. No hospitals or health systems have achieved consistent excellence throughout their institutions. High-reliability science is the study of organizations in industries like commercial aviation and nuclear power that operate under hazardous conditions while maintaining safety levels that are far better than those of health care. Adapting and applying the lessons of this science to health care offer the promise of enabling hospitals to reach levels of quality and safety that are comparable to those of the best high-reliability organizations.

**Methods:**

We combined the Joint Commission's knowledge of health care organizations with knowledge from the published literature and from experts in high-reliability industries and leading safety scholars outside health care. We developed a conceptual and practical framework for assessing hospitals’ readiness for and progress toward high reliability. By iterative testing with hospital leaders, we refined the framework and, for each of its fourteen components, defined stages of maturity through which we believe hospitals must pass to reach high reliability.

**Findings:**

We discovered that the ways that high-reliability organizations generate and maintain high levels of safety cannot be directly applied to today's hospitals. We defined a series of incremental changes that hospitals should undertake to progress toward high reliability. These changes involve the leadership's commitment to achieving zero patient harm, a fully functional culture of safety throughout the organization, and the widespread deployment of highly effective process improvement tools.

**Conclusions:**

Hospitals can make substantial progress toward high reliability by undertaking several specific organizational change initiatives. Further research and practical experience will be necessary to determine the validity and effectiveness of this framework for high-reliability health care.

Almost fourteen years have passed since the institute of Medicine's report “To Err Is Human” galvanized a national movement to improve the quality and safety of health care (Kohn, Corrigan, and Donaldson 2000). Isolated examples of improvement now can be found, and some of the results are impressive (Dixon-Woods et al. 2011; Pronovost et al. 2006, 2010). Measured against the magnitude of the problems, however, the overall impact has been underwhelming. Every year, millions of people suffer the adverse effects of health care–associated infections and harmful medication errors (Aspden et al. 2007; Klevans et al. 2007). More people are harmed by errors during transitions from one health care setting to another (Bodenheimer 2008; Forster et al. 2003). Operations on the wrong patient or the wrong body part continue to take place, perhaps as often as fifty times per week in the United States (estimated from: Minnesota Department of Health 2013). Fires break out in our operating rooms during surgery, perhaps as frequently as six hundred times a year, often seriously injuring the patient (ECRI Institute 2013).

The frequency and severity of these failings stand in particularly sharp contrast to the extraordinary successes that industries outside health care have had in achieving and sustaining remarkable levels of safety. Commercial air travel, nuclear power, and even amusement parks are pertinent examples. Organizations in these and other industries have been the subject of scholarly study, seeking to understand what characteristics and behaviors create the conditions that produce such exemplary performance or “high reliability” (Reason 1997; Weick and Sutcliffe 2007). Could health care become highly reliable as well? What would health care organizations have to do differently to achieve this goal? To address these questions, a team at the Joint Commission has worked closely with experts in high reliability from academia and industry. We combined this knowledge with an understanding of health care quality and safety that derives from our daily work with the more than 20,000 U.S. health care organizations that we accredit or certify. In an earlier article, we discussed the historical context of the challenge of high-reliability health care and described the broad outlines of a conceptual framework that might enable health care organizations to chart a path toward high reliability (Chassin and Loeb 2011). In this article, we report the further elaboration of that work in the form of a practical framework that individual health care organizations can use to evaluate their readiness for and progress toward the goal of high reliability. The framework describes four stages of maturity that define milestones on this road for each of fourteen specific characteristics of health care organizations. Although some elements of this framework may be relevant to many different kinds of health care organizations, we developed it specifically for hospitals. Indeed, the most serious problems with the quality of health care are found in hospitals, and some hospitals are already working toward becoming highly reliable.

## What Can High-Reliability Organizations Teach Health Care?

We began our investigation of what high reliability might mean for health care by analyzing what is known about how highly reliable organizations function. Weick and Sutcliffe provide the most compelling depiction of how high-reliability organizations (HROs) stay safe. They describe an environment of “collective mindfulness” in which all workers look for, and report, small problems or unsafe conditions before they pose a substantial risk to the organization and when they are easy to fix (Weick and Sutcliffe 2007). These organizations rarely, if ever, have significant accidents. They prize the identification of errors and close calls for the lessons they can extract from a careful analysis of what occurred before these events. These lessons point to specific weaknesses in safety protocols or procedures that can be remedied to reduce the risk of future failures.

The five high-reliability principles that Weick and Sutcliffe spell out further elucidate the capability of high-reliability organizations to achieve and maintain exemplary levels of safety. HROs are preoccupied with failure, never satisfied that they have not had an accident for many months or many years, and they are always alert to the smallest signal that a new threat to safety may be developing. People who work in HROs also resist the temptation to simplify their observations and their experiences of their environment. Threats to safety can be complex and present themselves in many different forms. Accordingly, being able to identify the often subtle differences among threats may make the difference between early and late recognition—between finding an unsafe condition when it is easy to correct and failing to recognize a problem until it is getting out of control. The third principle of high reliability is sensitivity to operations. HROs recognize that the earliest indicators of threats to organizational performance typically appear in small changes in the organization's operations. They thus take great pains to ensure that all those workers who are most intimately involved in operations always report any deviations from expected performance. In addition, HROs make sure that everyone not only feels free to speak up with any concerns but also recognizes an obligation to do so because of how highly the organization values the information as a vital component of its ability to achieve its highest priority: near-perfect safety.

The fourth principle is commitment to resilience. HROs recognize that despite all their best efforts and past safety successes, errors will occur and safety will be threatened. “The hallmark of an HRO is not that it is error-free but that errors don't disable it” (Weick and Sutcliffe 2007, 14). Resilience refers to an organization's capability to recognize errors quickly and contain them, thereby preventing the harm that results when small errors propagate, are compounded, and mushroom into major problems. HROs enhance their resilience by adhering to the fifth principle: deference to expertise. When confronted by a new threat, HROs have mechanisms in place to identify the individuals with the greatest expertise relevant to managing the new situation and to place decision-making authority in the hands of that person or group. They do not invoke organizational hierarchy or expect that the person with the most seniority or highest rank will be the most effective at dealing with the problem.

## Assessing Hospitals’ Current Performance against the Principles of High Reliability

How close or far away is the typical hospital today from this state of high reliability? The answer is, quite far. In health care, we rarely observe the five principles of high reliability guiding the actions of organizations, their leaders, and caregivers. As opposed to a preoccupation with avoiding failure, hospitals and other health care organizations behave as if they accept failure as an inevitable feature of their daily work. How else could we explain the estimates that 99,000 Americans die in hospitals each year from health care–associated infections while hand hygiene compliance routinely registers in the 40 percent range—among many other examples? (Erasmus et al. 2010; Klevans et al. 2007). Operations on the wrong body part or the wrong patient should never occur. Neither should fires during surgery. Fortunately, these events happen only rarely—from the perspective of an individual surgeon or hospital—but we are not close to eliminating them entirely from American health care. In health care, the rarity of adverse events like these tends to reinforce organizations’ beliefs that they will never experience them and leads to a misplaced confidence that their safety systems are adequate. This complacency blunts the alertness of surgical teams to the small signs of a risk of a surgical fire or wrong-site surgery. HROs recognize that complacency itself is a threat to safety and so take great pains to not let it take root.

Failing to resist the temptation to simplify frequently impedes safety efforts in health care. For example, we often approach a quality problem with a simple, one-size-fits-all “best practice” solution. The Joint Commission's Universal Protocol, developed to eliminate wrong-site surgery, is one such example. It consists of three simple steps: (1) verify the identity of the patient and the intended procedure, (2) mark the surgical site, and (3) conduct a “time-out” in the operating room just before the surgery begins in order to verify again that the patient, the procedure, and the operative site are correctly identified. But this overly simple approach has not eliminated the problem, in large part because it fails to account for the complexities of the surgical process and all the different ways in which risks of a wrong-site procedure may be introduced into it. For example, such risks may arise while scheduling the surgical procedure, a set of problems that the Universal Protocol does not address.

One of the most pervasive safety problems in hospitals relates to their failure to be sensitive to operations. Health care workers at all levels routinely observe unsafe conditions, behaviors, and practices, but they very often fail to bring those problems to the attention of managers who are placed appropriately in the daily work flow to address the problems quickly. Several factors contribute to this gap. Poor communication both within and between teams is a common condition in health care. Transitions from one care setting to another (so-called handoffs) are fraught with the risk of error due to the incomplete or inaccurate communication of crucial patient information. When caregivers come to expect poor communication, they become desensitized to its hazards. In one analysis, such a “culture of low expectations” explained a substantial number of the errors that led to a patient's undergoing an invasive procedure that was intended for someone else (Chassin and Becher 2002). Thus, the lack of recognition of unsafe conditions or practices is one important reason they are not reported.

In addition, health care workers of all kinds are exposed to an inordinate amount of intimidating behavior that suppresses their reporting of safety problems. Physicians are often seen as the initiators of intimidating or disrespectful behavior, and nurses are commonly seen as its targets (Leape et al. 2012; Saxton, Hines, and Enriquez 2009). But caregivers of all kinds are involved in these unsafe situations. In 2004, the Institute for Safe Medication Practices published the results of its Workplace Intimidation Survey, which focused on the process of receiving, interpreting, and acting on medication orders (Institute for Safe Medication Practices 2004). More than two thousand respondents, mainly nurses and pharmacists, reported a variety of these behaviors that they had personally experienced in the preceding twelve months. The most common behaviors perceived as intimidating were not the flagrantly abusive practices of throwing objects or using loud or profane language. Rather, the failure to return phone calls or pages, the use of condescending language, and impatience with questions topped the list. Between 60 and 67 percent of the respondents said they had personally experienced such behaviors initiated by physicians three or more times in the preceding year, and 20 to 28 percent said that they had experienced those behaviors more than ten times. About half those numbers reported experiencing the same behaviors by nonphysicians. The caregivers who experienced these behaviors employed a variety of strategies—all suboptimal and risky—to deal with them, including asking someone else to talk to an intimidating prescriber about a safety concern regarding a medication order (39%), refraining from contacting the prescriber while attempting to clarify the safety of a drug order on their own (67%), or asking colleagues to help interpret an order to avoid having to interact with a particular prescriber (75%). HROs do not tolerate the existence of intimidating behaviors that suppress the reporting of safety concerns and perpetuate the existence of unsafe conditions.

A specific example helps illuminate the complexities of the barriers that hospitals face in trying to be sensitive to these safety signals. Many medical devices employed in routine hospital care come equipped with alarms that make various sounds when preset parameters are exceeded. Intravenous infusion pumps, cardiac rate and rhythm monitors, mechanical ventilators, and blood oxygen monitors are some of the more common ones. Caregivers are bombarded hourly by these alarms, especially those working in intensive care areas housing the sickest patients with the greatest number of devices per patient. The number of alarms that sound per patient per day can total several hundred. For a variety of reasons, the vast majority (perhaps as many as 85% to 99%) of these alarm sounds do not signify clinical situations of danger. These reasons include poor integration of devices with one another, equipment malfunction, inappropriate alarm settings, and gaps in staff training. The result is that caregivers experience “alarm fatigue” and may take a variety of unsafe actions, such as turning off the alarms entirely, turning down the sound volume to the point of inaudibility, resetting the alarm to unsafe levels, or ignoring the alarm sounds altogether (Joint Commission 2013). If this sounds like a dangerous mix of unsafe conditions, it is. The Joint Commission's voluntary adverse event reporting program recorded ninety-eight alarm-related events between 2009 and June 2012, with eighty of them resulting in death. The ECRI Institute has cited this problem as one of the top ten health technology hazards each year since 2007 (ECRI Institute 2012). A comprehensive solution to this problem would require many stakeholders to work together, including device manufacturers, information technology experts, physicians, medical informatics professionals, nurses, clinical engineers, and hospital administrators. Imagine the risks to safety if a nuclear power plant had alarm systems that functioned in this fashion. No HRO would permit a condition this unsafe to exist.

Hospitals and health care organizations do not exhibit the features of resilience that characterize HROs. In a high-reliability environment, errors and unsafe conditions are recognized early and prevented by rapid remediation from causing harm. But in health care, uncoordinated and poorly designed and maintained mechanical systems (like medical device alarms) are tolerated, even though they are not safe. Intimidating behaviors suppress reporting and lead to additional unsafe behaviors as caregivers create workarounds to avoid repetitive exposure to intimidators. Errors are not seen as valuable information, essential to a hospital's ability to improve patient safety. In its 2012 report of the results of its annual patient safety culture survey, the federal Agency for Healthcare Research and Quality stated that on average, 65 percent of respondents from 1,128 hospitals worried that mistakes they had made were kept in their personnel files, and 50 percent agreed that staff felt that their mistakes were held against them (Agency for Healthcare Research and Quality 2012).

Finally, in attempting to solve safety and quality problems, hospitals do not regularly permit the most expert individual to implement solutions. Instead, multiple hierarchies dominate the authority structures of most hospitals. Senior physicians often disregard the observations of their juniors. Nursing hierarchies can be as rigid as those of their physician colleagues. Pharmacists often have a difficult time bringing their considerable expertise to bear to avoid medication errors. Too often, health care teams are multidisciplinary in name only, with physicians or senior administrators dominating the scene. The “fallacy of centrality” is frequently on display in hospitals. Westrum coined this term during a sociological analysis of why pediatricians failed to identify child abuse until the 1960s. He suggested that one of the important underlying phenomena was pediatricians’ ingrained belief that they were “central” to all issues relevant to children's health. This mind-set of “centrality” has adverse consequences. If something as crucial to a child's health as physical abuse by a parent were going on, surely pediatricians would know about it and bring it to the attention of other pediatricians. But they didn't know about it, and therefore it wasn't happening (Westrum 1982). In health care, the risk of an individual's falling prey to the fallacy of centrality would seem to increase with seniority. This mind-set is particularly risky for organizational leaders because it encourages the risky belief that “no news is good news.” In hospitals, “no news” most often means that intimidated caregivers are not recognizing or reporting the unsafe conditions that will, soon enough, harm patients. Thus, available data and considerable experience suggest strongly that the five principles of high reliability would be unrecognizable in an average hospital's daily work. To the contrary, in several instances, particularly those involving the rapid identification and management of errors and unsafe conditions, it appears that today's hospitals often exhibit the very opposite of high reliability.

There is an important corollary to the observation that hospitals are currently characterized by low reliability. This fact implies strongly that hospitals cannot solve these problems by simply and directly adopting high-reliability principles and practices all at once. Imagine what might happen if all the workers in a hospital suddenly acquired a keen sense of collective mindfulness and began to recognize and report all the unsafe conditions and errors they encountered from the moment they arrived at the hospital. The organization would soon be so deluged with such reports that its capacity to fix the problems uncovered by the reports would be overwhelmed, and many unsafe conditions would necessarily remain unaddressed. Of course, such a transformation of an organization's culture cannot take place over night. But that is precisely the point. We must take careful note of how hospitals function today in all the key arenas that must change if high reliability is to become possible for them. This possibility will become more real if we can accurately describe hospitals’ current state and chart a plausible and feasible pathway toward high reliability, one that defines specific milestones representing incremental progress.

Is there any guidance in the high-reliability literature on how to chart such a pathway? Not much. Weick and Sutcliffe offer a series of “audits” or rating scales that assess the extent to which an organization is behaving like an HRO and give some general advice about how to improve (Weick and Sutcliffe 2007, chaps. 5 and 6). But these tools are not specific to health care. Reason offers a similar assessment tool, his “Checklist for Assessing Institutional Resilience (CAIR),” and has adapted it for health care (Carthey, de Leval, and Reason 2001). These thoughtful contributions help focus us on what hospitals should be doing to become highly reliable. But they do not give us much insight into precisely how these goals can be accomplished. In brief, we know of no well-documented blueprints for elevating a low-reliability organization or industry into a highly reliable one and sustaining that achievement over time.

## Adapting High-Reliability Science to Hospitals

As noted earlier, we described elsewhere a broad conceptual framework for adapting high-reliability science to health care organizations (Chassin and Loeb 2011). This framework was derived from the integration of high-reliability science, our considerable experience working with the thousands of health care organizations that the Joint Commission accredits or certifies, and some studies explaining how some hospitals have started to adapt high-reliability principles to their operations (Dixon and Shofer 2006; Fei and Vlasses 2008; Frankel, Leonard, and Denham 2006; May 2013; Wolterman and Shabot 2012). We explored three major changes that health care organizations would have to undertake in order to make substantial progress toward high reliability: (1) the leadership's commitment to the ultimate goal of zero patient harm, (2) the incorporation of all the principles and practices of a safety culture throughout the organization, and (3) the widespread adoption and deployment of the most effective process improvement tools and methods. We elaborate here these three changes with specific respect to hospitals and health systems.

By leadership commitment, we mean the aligned agreement of the governing body, typically a board of trustees or directors, senior management, and physician and nurse leaders. All the constituencies of leadership, both formal and informal, must share the same singular vision of eventually eliminating harms to patients. This is an essential initial requirement, because the success of all the other changes depends on it. The goal of zero also is important because one of the most salient characteristics of high-reliability organizations is that they are not satisfied with whatever their current level of safety might be. They always are looking for ways to improve it. For example, the U.S. airline industry was extraordinarily safe during the 1990s. From 1990 through 2001, U.S. commercial aviation averaged 129 deaths per year from accidents and logged an average of 9.3 million flights per year, translating into a death rate of 13.9 deaths per million flights. In the next decade, however, from 2002 to 2011, that death rate plummeted by a remarkable 88 percent to 1.6 deaths per million flights. Even though the average annual number of flights increased to 10.4 million per year, the number of deaths dropped to an average of 16.6 per year (U.S. Department of Transportation 2012). The lesson for health care is not to be satisfied with modest improvements. Aiming for zero harm is the first step toward achieving it.

For the past thirty years, commercial aviation has invested heavily in radically changing flight crews’ culture in order to advance airline safety. This work began following research conducted by the National Aeronautics and Space Administration in the 1970s demonstrating that the majority of airplane crashes were caused not by catastrophic mechanical failures but by failures of communication among pilots and crew. The development and worldwide deployment of focused and highly effective training programs to establish a safety culture on aircraft flight decks followed. These programs, known as Crew Resource Management, are widely credited with playing the most important role in the dramatic safety improvements the industry witnessed over this time period (Helmreich, Merritt, and Wilhelm 1999). One of the original developers of Crew Resource Management for the airline industry has since turned his attention to health care. He and his colleagues found that the professional culture in operating rooms and the communication errors related to it were quite similar to those found among aircraft crews (Helmreich 2000). This work has led to a series of efforts to apply the principles and methods of Crew Resource Management to health care (Gordon, Mendenhall, and O'Connor 2013).

Since 2009, the Joint Commission has required the leadership of all health care organizations that it accredits to “create and maintain a culture of safety” (Joint Commission 2008a). Consequently, many hospitals now conduct staff surveys to assess their safety culture (Agency for Healthcare Research and Quality 2012; Sexton et al. 2006). Few, however, have moved beyond tabulating survey results to taking effective actions that have resulted in creating the kind of safety culture that supports high reliability. We have few proven tools or methods that can guide hospital leaders to achieve a fully functional safety culture. The model we describe in our practical framework is derived from the work of Reason and Hobbs (Reason and Hobbs 2003). The organizational culture they depict is based on Reason's years of studying complex organizations and how they prevent or fail to prevent accidents that cause harm. We believe that this model is the one most adaptable and appropriate to health care.

The third of the major changes relates to how hospitals carry out efforts to improve the performance of their care processes. It is in this domain that high-reliability science provides the least guidance to health care. HROs do not have safety processes that fail 40 to 60 percent of the time, which is the case in health care (e.g., hand hygiene and handoff communication) (Bodenheimer 2008; Erasmus et al. 2010). The specific tools and methods that HROs use to maintain their nearly perfect safety procedures are not directly relevant in a setting with reliability as low as that of health care. So we must look elsewhere. We believe that three sets of process improvement tools—lean, six sigma, and change management—constitute the most effective way for health care to dramatically enhance its capacity to create nearly perfect safety processes (DelliFraine, Langabeer, and Nembhard 2010; DuPree et al. 2009). We call the three “robust process improvement,” or RPI (Joint Commission Center for Transforming Healthcare 2013). They represent the next generation of process improvement methods that were developed in industry and imported into health care. They are proving to be far more effective in addressing complex clinical quality and safety problems than PDCA (“plan, do, check, act”) or their more immediate predecessors (continuous quality improvement and total quality management) (Goldberg 2000). One of the most important distinguishing features of these newer improvement methods is their systematic attention to uncovering all the very specific causes of the failures of safety processes (e.g., hand hygiene). By pinpointing specific causes (e.g., improper use of gloves or faulty maintenance procedures that do not keep hand gel dispensers full) and by measuring which ones are most prevalent in a particular area of a hospital, these tools direct improvement efforts to eliminate the causes of the majority of failures. By their careful attention to unraveling the complexities of health care quality and safety problems, the tools of robust process improvement offer health care the means to implement the “reluctance to simplify” principle of high reliability.

The Joint Commission has adopted RPI as its internal process improvement methodology and, in the first five years of the program, which began in 2008, trained 35 percent of its workforce in using these tools (Adrian 2009). Since 2009, the Joint Commission's Center for Transforming Healthcare has been applying these RPI tools together with teams from hospitals around the country that also have mastered their use to address a number of health care's most persistent quality and safety problems. We have found them to be highly effective. Table 1 shows the rates of improvement demonstrated in the Center's first four projects. The Joint Commission's experience is consistent with that of companies such as GE that have employed the same tools for many years with great benefit (Bartlett and Wozny 2005; Rao 2011).

**TABLE 1:** Improvements Seen in Four Projects Using Robust Process Improvement

Problem Addressed	Number and Type of Health Care Organizations	Measure	Before (%)	After (%)	Relative Improvement (%)
Hand hygiene	8 hospitals	Hand hygiene compliance^a^	47.5	81	71 *p* = 0.000
Handoff communication	10 hospitals	Ineffective handoffs at care transitions^b^	41	18	56 *p* = 0.007
Wrong-site surgery risks	5 hospitals, 3 ambulatory surgery centers	Risk of wrong-site surgery^c^			
Scheduling			39	21	46 *p* = 0.000
Preoperative area			52	19	63 *p* = 0.000
Operating room			59	29	51 *p* = 0.000
Colorectal surgical-site infections (SSI)	7 hospitals	Cases with an SSI^d^	15.8	10.7	32 *p* = 0.000

*Notes:* Robust Process Improvement is a combination of three complementary process improvement methods: lean, six sigma, and change management.

a Percentage of times that caregivers cleaned their hands before walking into or out of a patient's room.

b Percentage of handoffs that failed to provide complete information necessary to patient care.

c Percentage of cases with any risk of wrong-site surgery.

d Percentage of colorectal surgery cases with any surgical-site infection.

*Source*: http://www.centerfortransforminghealthcare.org/projects/projects.aspx.

## The High-Reliability Health Care Maturity Model: A Practical Framework for Improvement

Having established these three major domains of change, we then considered how hospitals and health systems operate today and how they might evolve (slowly or rapidly) toward high reliability in each of these three areas. Clearly, the industry contains much heterogeneity, but observing those differences helps better characterize the current state and directions for that evolution. In devising this framework, we identified several specific components of each of the three domains of change (fourteen components in all) and four stages of maturity for each of them that would define progress toward high reliability. We depicted the four stages as beginning, developing, advancing, and approaching. We observed hospitals or health systems in which a few or several of these components currently reside in each of these four stages of high reliability. But we intentionally did not attempt to add a fifth stage (perhaps to be labeled “arriving” in the future) that would describe a high-reliability hospital, because we know of no hospitals that have achieved high reliability across all their activities and, therefore, have no firsthand observations to use for such a description.

We created this framework over a two-year period, employing a variety of methods and sources. A team at the Joint Commission has been engaged with high-reliability experts from academia and industry since 2010 to assimilate what is known about HROs with the institutional knowledge we have gained from our work in health care quality and safety. These experts include widely published authors and officials and executives from HROs in commercial aviation, the chemical and petroleum industries, nuclear power, and the military. Among other activities, the Joint Commission hosted the Fifth International High-Reliability Conference in May 2012, at which health care executives interacted with representatives from academia and HROs from ten different industries (Joint Commission 2012).

To produce the first draft of the framework, we combined the information gleaned from these experiences with the empirical literature on the characteristics of hospitals associated with improved safety and quality. We then conducted two rounds of pilot testing with health care leaders. In the first round, a small group of five individuals with hospital leadership roles as chief quality officers, chief medical officers, or chief executive officers examined the framework and provided qualitative assessments of its face validity, including whether all appropriate elements were included in the framework and whether any should be eliminated or defined differently. Separately, we produced for their review a self-assessment questionnaire designed to elicit information from hospital leaders that would permit us to assign each of the fourteen components of high reliability to one of the four stages of maturity. The first-round reviewers also assessed that instrument. Based on this first round of reviews, we made appropriate changes in the framework and the questionnaire.

In the second round of testing, we asked the leadership teams of seven U.S. hospitals to test the framework by using the questionnaire to assess their own hospitals. Each team engaged in this process four to six leaders from a variety of different leadership perspectives, which included chief executive officers, chief nursing officers, chief medical officers, chief quality officers, chief information officers, and others with similar responsibilities. We compiled the data from this round of testing and convened a face-to-face meeting with the teams’ leaders, typically the chief executive officer, of each of the seven hospitals to discuss the experiences of their teams. The results of this round of testing were incorporated into the framework and questionnaire, which were finalized for further field-testing. The framework is described in detail in the following sections.

### Leadership

Table 2 depicts the six components of leadership and each one's characteristics in the four stages of maturity. The six components are the board of trustees, the chief executive officer and all senior management (including nursing leaders), the engagement of physicians, the hospital's quality strategy, its use and dissemination of data on measures of quality, and the use of information technology to support quality and safety improvement. The identification of these specific components is supported by published literature linking them to better quality performance (Goeschel, Wachter, and Pronovost 2010; Jha and Epstein 2010; Weiner, Shortell, and Alexander 1997). The hospital leaders’ commitment to high reliability must include a prominent role for the board of trustees or directors. The board must be part of the leadership's commitment to eventually achieve zero patient harm and to elevate quality and patient safety to the organization's highest strategic goal. If the board is left out, management will find its efforts unsupported or misunderstood. Today, hospital boards vary over a wide spectrum of involvement in the quality programs of the hospitals they oversee (Jha and Epstein 2010).

**TABLE 2:** Leadership and High Reliability: Stages of Organizational Maturity

Leadership	Beginning	Developing	Advancing	Approaching
Board	Board's quality focus is nearly exclusively on regulatory compliance.	Full board's involvement in quality is limited to hearing reports from its quality committee.	Full board is engaged in the development of quality goals and approval of a quality plan and regularly reviews adverse events and progress on quality goals.	Board commits to the goal of high reliability (i.e., zero patient harm) for all clinical services.
CEO/management	CEO/management's quality focus is nearly exclusively on regulatory compliance.	CEO acknowledges need for plan to improve quality and delegates the development and implementation of a plan to a subordinate.	CEO leads the development and implementation of a proactive quality agenda.	Management aims for zero patient harm for all vital clinical processes; some demonstrate zero or near-zero rates of harm.
Physicians	Physicians rarely lead quality improvement activities; overall participation by physicians in these activities is low.	Physicians champion some quality improvement activities; physicians participate in these activities in some areas but not widely.	Physicians often lead quality improvement activities; physicians participate in these activities in most areas, but some important gaps remain.	Physicians routinely lead clinical quality improvement activities and accept the leadership of other appropriate clinicians; physicians’ participation in these activities is uniform throughout the organization.
Quality strategy	Quality is not identified as a central strategic imperative.	Quality is one of many competing strategic priorities.	Quality is one of the organization's top three or four strategic priorities.	Quality is the organization's highest-priority strategic goal.
Quality measures	Quality measures are not prominently displayed or reported internally or publicly; the only measures used are those required by outside entities and are not part of reward systems.	Few quality measures are reported internally; few or none are reported publicly and are not part of reward systems.	Routine internal reporting of quality measures begins, with the first measures reported publicly and the first quality metrics introduced into staff reward systems.	Key quality measures are routinely displayed internally and reported publicly; reward systems for staff prominently reflect the accomplishment of quality goals.
Information technology	IT provides little or no support for quality improvement.	IT supports some improvement activities, but principles of safe adoption are not often followed.	IT solutions support many quality initiatives; the organization commits to principles and the practice of safe adoption.	Safely adopted IT solutions are integral to sustaining improved quality.

In addition, physicians are essential to the success of any quality initiative in hospitals. Table 2 identifies two vital components of physicians’ role: leadership and participation. In order to move effectively toward high reliability, physicians must routinely champion quality improvement initiatives throughout the hospital. Both the formally appointed leaders (chief medical officer, vice president for medical affairs) and the informal leaders (medical staff president, voluntary medical staff leaders) need to be visible and active enthusiasts for quality, including physician leaders who are not employees of the hospital. Hospital leaders must decide on the organization's quality strategy. It is difficult to imagine a hospital getting close to high reliability if quality is merely one of many competing priorities. Memorial Hermann Health System, a twelve-hospital health care system based in Houston, has explicitly committed to becoming highly reliable and has specified the importance of all the major ingredients in this framework to their efforts (Shabot et al. 2013). As Dan Wolterman, the system's CEO pointed out, “Ensuring patient safety is our core value, and it's our only core value” (Wolterman and Shabot 2012). To accelerate the progress toward zero harm, quality must be measured and data on those measures must be widely available both within the hospital and to the public. Not only is such transparency valuable in its own right, but public reporting also is a powerful added force that drives improvement. The quality program and its measures should focus on meeting the needs and addressing the specific quality problems of the hospital's patient population. Other incentives, such as the judicious use of financial rewards and staff advancement opportunities based on performance against quality measures, are important accelerants as well. Finally, leaders are obligated to employ health information technology (IT) effectively in the service of quality improvement. IT is particularly important to an HRO, because it is frequently the vehicle by which nearly perfect processes sustain their performance. If a process has been so effectively redesigned as to be highly reliable, automating it is the most effective way to maintain it in that state. Unfortunately, in health care, automation is often deployed unsafely, a phenomenon that increases rather than decreases the risk of harm (Ash et al. 2007, 2009; Joint Commission 2008b; Koppel et al. 2005; Sparnon and Marella 2012). In addition, various types of health IT are often not coordinated, thereby increasing risk. For example, if programmable infusion devices are not supported by the same decision support rules that govern pharmacy systems and physician order entry systems, the resulting confusion can be life threatening for patients. A hospital approaching high reliability adopts health IT solutions in a coordinated and integrated manner following the principles of safe adoption (Joint Commission 2008b; Karsh 2004).

### Safety Culture

Table 3 shows the five components of safety culture and their manifestations in each of the four stages of maturity toward high reliability. A culture of safety that fully supports high reliability has three central attributes: trust, report, and improve (Reason and Hobbs 2003). Workers exhibit enough trust in their peers and the organization's management that they routinely recognize and report errors and unsafe conditions. This trust is established when the organization eliminates intimidating behavior that suppresses reporting, acts in a timely way to fix the problems reported by workers, and communicates these improvements consistently to the individuals who reported the problems in the first place. That communication in turn strengthens the trust that led to the reports and fosters further identification and reporting of problems even further upstream from harm. When all three of these components of a safety culture (trust, report, and improve) are working well, they reinforce one another and produce a stable organizational culture that sustains high reliability.

**TABLE 3:** Safety Culture and High Reliability: Stages of Organizational Maturity

Safety Culture	Beginning	Developing	Advancing	Approaching
Trust	Trust or intimidating behavior is not assessed.	First codes of behavior are adopted in some clinical departments.	CEO and clinical leaders establish a trusting environment for all staff by modeling appropriate behaviors and championing efforts to eradicate intimidating behaviors.	High levels of (measured) trust exist in all clinical areas; self-policing of codes of behavior is in place.
Accountability	Emphasis is on blame; discipline is not applied equitably or with transparent standards; no process exists for distinguishing “blameless” from “blameworthy” acts.	The importance of equitable disciplinary procedures is recognized, and some clinical departments adopt these procedures.	Managers at all levels accord high priority to establishing all elements of safety culture; adoption of uniform equitable and transparent disciplinary procedures begins across the organization.	All staff recognize and act on their personal accountability for maintaining a culture of safety; equitable and transparent disciplinary procedures are fully adopted across the organization.
Identifying unsafe conditions	Root cause analysis is limited to adverse events; close calls (“early warnings”) are not recognized or evaluated.	Pilot “close call” reporting programs begin in few areas; some examples of early intervention to prevent harm can be found.	Staff in many areas begin to recognize and report unsafe conditions and practices before they harm patients.	Close calls and unsafe conditions are routinely reported, leading to early problem resolution before patients are harmed; results are routinely communicated.
Strengthening systems	Limited or no efforts exist to assess system defenses against quality failures and to remedy weaknesses.	RCAs begin to identify the same weaknesses in system defenses in many clinical areas, but systematic efforts to strengthen them are lacking.	System weaknesses are cataloged and prioritized for improvement.	System defenses are proactively assessed, and weaknesses are proactively repaired.
Assessment	No measures of safety culture exist.	Some measures of safety culture are undertaken but are not widespread; little if any attempt is made to strengthen safety culture.	Measures of safety culture are adopted and deployed across the organization; efforts to improve safety culture are beginning.	Safety culture measures are part of the strategic metrics reported to the board; systematic improvement initiatives are under way to achieve a fully functioning safety culture.

Maintaining trust also requires the organization to hold employees accountable for adhering to safety protocols and procedures. HROs establish clear, equitable, and transparent processes for recognizing and separating the small, blameless errors that all people make every day from unsafe or reckless actions that are blameworthy. Understanding how and why blameless errors occur is part of the learning process that HROs employ to maintain their exemplary safety records. Recognizing and dealing appropriately with blameworthy acts is an equally important dimension of an HRO's safety culture because of its vital role in maintaining trust. Unfortunately, health care organizations too often punish staff for blameless acts while failing to implement equitable disciplinary procedures for those who commit blameworthy acts. Nor have hospital leaders succeeded in eradicating intimidating behaviors (Institute for Safe Medication Practices 2004). These failings explain the lack of trust among hospital staff noted earlier (Agency for Healthcare Research and Quality 2012). Hospitals that move toward high reliability establish codes of behavior that are modeled by leaders (including nurses and physicians) who champion efforts to eliminate intimidation and encourage and reward the reporting of blameless errors and unsafe conditions. Accountability for adhering to safe practices should be ingrained in all employees and is spurred by implementing standards for invoking disciplinary procedures that apply to all staff, regardless of seniority or professional credentials. For example, Maimonides Medical Center in New York City has established such a program in its Code of Mutual Respect, which commits all stakeholders (including physicians, nurses, staff, students, vendors, consultants, and volunteers) to “working harmoniously” together and to eliminate intimidating behaviors. The program includes progressive interventions, including disciplinary actions for individuals who repeatedly violate the code (Maimonides Medical Center 2009).

HROs also proactively assess the strength and resilience of their safety systems and the organizational defenses that prevent errors from propagating and leading to harm. Today's hospitals function in primarily a reactive mode, investigating incidents in which patients have already been harmed, conducting root cause analyses, and instituting corrective action plans to prevent future occurrences. Becoming much safer requires caregivers’ willingness and ability to recognize and report close calls and unsafe conditions, combined with an organizational capacity to act effectively on those reports to eliminate the risks they embody. Furthermore, as opposed to today's norm of focusing on single events, hospitals should compile the results of their investigations across many harm events and close calls to identify which of their safety systems or defenses are most in need of improvement. These evaluations should lead to the development of proactive assessments of key safety systems (e.g., those that relate to medication administration and infection prevention and control) so that weaknesses can be identified and remedied before they pose any significant risk to patients.

Finally, progress toward establishing all these elements of a culture of safety should be measured. Today, many hospitals regularly use one of several available staff surveys to assess their safety culture. Few, however, analyze the meaning of the survey data, evaluate where each area of the hospital is falling short, and undertake specific, focused interventions to remedy those shortcomings. As hospitals make more progress toward high reliability, they will include safety culture metrics as part of their strategic planning programs, set goals for improving on those metrics, and report on those metrics to their boards, just as they report on metrics related to financial performance or patient satisfaction.

### Robust Process Improvement

Hospitals need new process improvement tools and methods to break out of their current state of low reliability. We have argued that robust process improvement (RPI)—a combination of lean, six sigma, and change management—is a much more potent set of tools than health care currently uses to address safety and quality problems. Briefly, and oversimplifying somewhat, *lean* is a set of tools and a philosophy of employee-empowered improvement that identifies and removes wasted effort from processes without compromising the quality of the outcome. *Six sigma* tools focus on improving the outcomes of a process by radically reducing the frequency with which defective products or outcomes occur. Lean and six sigma tools produce markedly improved processes. *Change management* is a systematic approach, used alongside lean and six sigma, that prepares an organization to accept, implement, and sustain the improved processes that result from the application of lean and six sigma tools. These three sets of tools are complementary, and together they provide the best available methods for hospitals to achieve major improvements in faulty processes.

Table 4 shows the three components of RPI and how each changes as a hospital comes closer to high reliability. Like GE, Best Buy, and other companies that have benefited from RPI, we believe that getting the most benefit from them requires that they be employed as a common language throughout the entire organization (Bartlett and Wozny 2005; Rao 2011). Nearly all employees should be trained at levels appropriate to each one's job. The tools should be used throughout the organization for all improvement work. Finally, proficiency in the use of RPI should be a part of every employee's performance appraisal and be required for career advancement within the organization. These elements provide vital support for spreading the use of these tools. For HROs, quality and safety are the personal responsibility of every employee, and being armed with highly effective ways to solve complex problems gives employees some of what they need to exercise that responsibility.

**TABLE 4:** Robust Process Improvement and High Reliability: Stages of Organizational Maturity

Performance Improvement	Beginning	Developing	Advancing	Approaching
Methods	Organization has not adopted a formal approach to quality management.	Exploration of modern process improvement tools begins.	Organization commits to adopt the full suite of Robust Process Improvement (RPI) tools.	Adoption of RPI tools is accepted fully throughout the organization.
Training	Training is limited to compliance personnel or to the quality department.	Training in performance improvement tools outside the quality department is recognized as critical to success.	Training of selected staff in RPI is under way, and a plan is in place to broaden training.	Training in RPI is mandatory for all staff, as appropriate to their jobs.
Spread	No commitment to widespread adoption of improvement methods exists.	Pilot projects using some new tools are conducted in a few areas.	RPI is used in many areas to improve business processes as well as clinical quality and safety; a positive ROI is achieved.	RPI tools are used throughout the organization for all improvement work; patients are engaged in redesigning care processes, and RPI proficiency is required for career advancement.

Today's hospitals generally lag far behind this ideal state. Some have used some of the elements of RPI, often starting with lean, but relatively few hospitals have adopted the full suite of RPI tools. Fewer still have engaged patients when using these powerful tools to redesign care processes. RPI provides highly effective tools for obtaining the “voice of the customer,” and the perspective of patients on what constitutes a high-quality outcome for a particular care process is vital to its improvement.

Making substantial progress toward high reliability in safety and quality requires the application of tools like RPI that can generate extremely high rates of sustainable improvement when applied to the poorly performing safety processes that exist in most hospitals today (see table 1). We know of no other approach to process improvement available at present that is capable of generating and sustaining rates of improvement of this magnitude consistently over the widest array of areas—from clinical quality to business processes (Chassin 1998; R. Chassin 2008).

### Future Research and Development Tasks

Enabling hospitals to use this high-reliability health care framework requires additional work. To advance toward high reliability, hospitals must be able to assess their current state of maturity with respect to each of the framework's fourteen components and then to access proven strategies, tools, and methods to advance to more mature levels. The Joint Commission is developing and testing an instrument that will permit hospital leaders to perform such an assessment. This instrument incorporates all the framework's elements with applicable definitions and measures. This instrument's psychometric properties will be the subject of future research. The utility of the assessment to hospitals in identifying their most pressing opportunities for making progress toward high reliability and the availability of specific tools to facilitate such progress is currently being field-tested. This initiative, the South Carolina Safe Care Commitment, is being led by the Joint Commission Center for Transforming Healthcare and the South Carolina Hospital Association (May 2013).

### Policy Implications

Those stakeholders with a vested interest in moving health care further and faster toward high reliability include state and federal government health agencies, consumer and patient advocacy groups, employers, public and private payers, health care professional organizations, and hospitals and health systems themselves. All have important roles to play in facilitating this transformation. Although a comprehensive assessment of these roles is beyond the scope of this article, several observations are nonetheless pertinent here. Regulatory mandates are unlikely to be effective in this effort. Regulation had only a modest and supportive role in the dramatic quality and safety improvements in other industries (e.g., commercial aviation, car manufacturing, and consumer electronics). In health care, regulators should pay attention first and foremost to identifying and eliminating requirements that obstruct progress toward high reliability. In some instances, such requirements impose outdated and ineffective methods of quality management. In others, they impose unproductive work on regulated organizations that distracts them from dealing more effectively with their quality challenges. Regulators can support the transformation to high reliability, for example, by well-crafted programs of publicly reporting reliable and valid measures of quality. Other U.S. industries have undergone transformations in quality stimulated primarily by competitive pressures (e.g., from Japanese automakers). A similar occurrence in health care is difficult to imagine because of the intensely local environment in which the large majority of hospitals and health systems operate (Becher and Chassin 2001).

Because the changes health care must undergo to become highly reliable are so thoroughgoing and profound, the primary drive for change must ultimately come from the health care organizations themselves. As the saying goes, it takes only one psychiatrist to change a lightbulb, but the lightbulb has to want to change. Many health care leaders are reluctant to commit to the goal of high reliability because they regard it as unrealistic or unachievable or a distraction from their current serious fiscal and regulatory pressures. One of the important roles for policymakers and stakeholders is to encourage, persuade, and demand that health care organizations embark on this journey. Even after they have committed to do so, how long it will take for health care organizations to reach high reliability is unknown, because none has arrived at that destination yet. Cincinnati Children's Hospital Medical Center has been working toward this goal for a more than a decade, and its current strategic plan calls for eliminating serious patient harm by 2015 (Cincinnati Children's Hospital Medical Center 2013).

Finally, hospitals and systems like Memorial Hermann and Cincinnati Children's that have been trailblazers in striving for high-reliability health care have developed their own strategies and tools, largely through trial and error. For this movement to broaden and deepen, the next wave of hospitals and health systems will need proven tools and methods to speed their journey through higher levels of maturity. Many stakeholders could contribute to the development and evaluation of such tools, and policymakers at several levels of government could facilitate this process by focused funding efforts.

## Conclusion

Achieving high reliability in health care will require hospitals to undergo substantial changes that cannot take place rapidly. We have outlined a framework, with fourteen components, for the practical application of these changes to hospitals. The components are distributed in three major domains: leadership, safety culture, and robust process improvement. We described for each component four evolutionary stages of maturity on the road to high reliability. Each stage provides hospitals with specific guidance on what actions they need to take in order to advance toward high reliability. Further research and experience derived from the application of this practical framework will be required to assess its effectiveness in facilitating hospitals’ advancement toward high reliability. Finally, policymakers and stakeholders in various positions should evaluate how they can support and accelerate this transformation.
